# Emerging trends in the etiology of enteric pathogens as evidenced from an active surveillance of hospitalized diarrhoeal patients in Kolkata, India

**DOI:** 10.1186/1757-4749-2-4

**Published:** 2010-06-05

**Authors:** Gopinath Balakrish Nair, Thandavarayan Ramamurthy, Mihir Kumar Bhattacharya, Triveni Krishnan, Sandipan Ganguly, Dhira Rani Saha, Krishnan Rajendran, Byomkesh Manna, Mrinmoy Ghosh, Keinosuke Okamoto, Yoshifumi Takeda

**Affiliations:** 1 National Institute of Cholera and Enteric Diseases (NICED), P-33, CIT Road, Beliaghata, Kolkata 700010, West Bengal, India; 2 Infectious Diseases and Beliaghata General Hospital, 59-Suren Sarker Road, Beliaghata, Kolkata 700037, West Bengal, India; 3 Graduate School of Medicine, Dentistry and Pharmaceutical Sciences, Okayama University, Okayama 700-8530, Japan; 4 Collaborative Research Center of Okayama University for Infectious Diseases in India, NICED, P-33, CIT Road, Beliaghata, Kolkata 700010, West Bengal, India

## Abstract

**Background:**

This study was conducted to determine the etiology of diarrhoea in a hospital setting in Kolkata. Active surveillance was conducted for 2 years on two random days per week by enrolling every fifth diarrhoeal patient admitted to the Infectious Diseases and Beliaghata General Hospital in Kolkata.

**Results:**

Most of the patients (76.1%) had acute watery diarrhoea in association with vomiting (77.7%) and some dehydration (92%). *Vibrio cholerae *O1, Rotavirus and *Giardia lamblia *were the important causes of diarrhoea. Among *Shigella *spp, *S. flexneri *2a and 3a serotypes were most predominantly isolated. Enteric viruses, EPEC and EAEC were common in children <5 year age group. Atypical EPEC was comparatively higher than the typical EPEC. Multidrug resistance was common among *V. cholerae *O1 and *Shigella *spp including tetracycline and ciprofloxacin. Polymicrobial infections were common in all age groups and 27.9% of the diarrhoea patients had no potential pathogen.

**Conclusions:**

Increase in *V. cholerae *O1 infection among <2 years age group, resistance of *V. cholerae *O1 to tetracycline, rise of untypable *S. flexnerii*, higher proportion of atypical EPEC and *G. lamblia *and polymicrobial etiology are some of the emerging trends observed in this diarrhoeal disease surveillance.

## Background

Global, regional and national estimates clearly place diarrhoeal diseases as a major, albeit a substantially neglected, public health problem. Deaths of children aged <5 years owing to diarrhoea was estimated to be 1.87 million at the global level (uncertainty range from 1.56 to 2.19 million), which is approximately 19% of total child deaths [1]. In the south-east Asian region, almost 48% of the estimated 3.07 million deaths annually are attributed to acute respiratory infections and diarrhoeal diseases with the highest burden of diarrhoeal disease in 5 countries: Bangladesh, India, Indonesia, Myanmar and Nepal where these diseases cause 60,000 deaths annually [2,3].

Diarrhoea is a syndrome that can be caused by different bacterial, viral and parasitic pathogens. Accurate understanding of the cause of diarrhoea in a given setting is an onerous task that requires systematic monitoring of the various pathogens. The availability of a well equipped clinical microbiology laboratory is a prerequisite to undertake such studies. Previous studies conducted at the National Institute of Cholera and Enteric Diseases (NICED), which includes hospital and community based surveillance for diarrhoea was focused on common enteric pathogens using conventional assays [3-5]. From November 2007, we expanded the scope of the hospital surveillance on diarrhoea by increasing the search for additional etiologies by the inclusion of campylobacters, different pathogroups of diarrhoeagenic *Escherichia coli *(DEC) and enhancing the search for enteric viruses and parasites using conventional, immunological and molecular detection methods. This report highlights the results on a systematic surveillance over two years for diarrhoeal etiologies in hospitalized patients at one of the largest Infectious Diseases Hospital in Southeast Asia located in Kolkata.

## Results

### Demography of enrolled subjects

**Figure 1:**
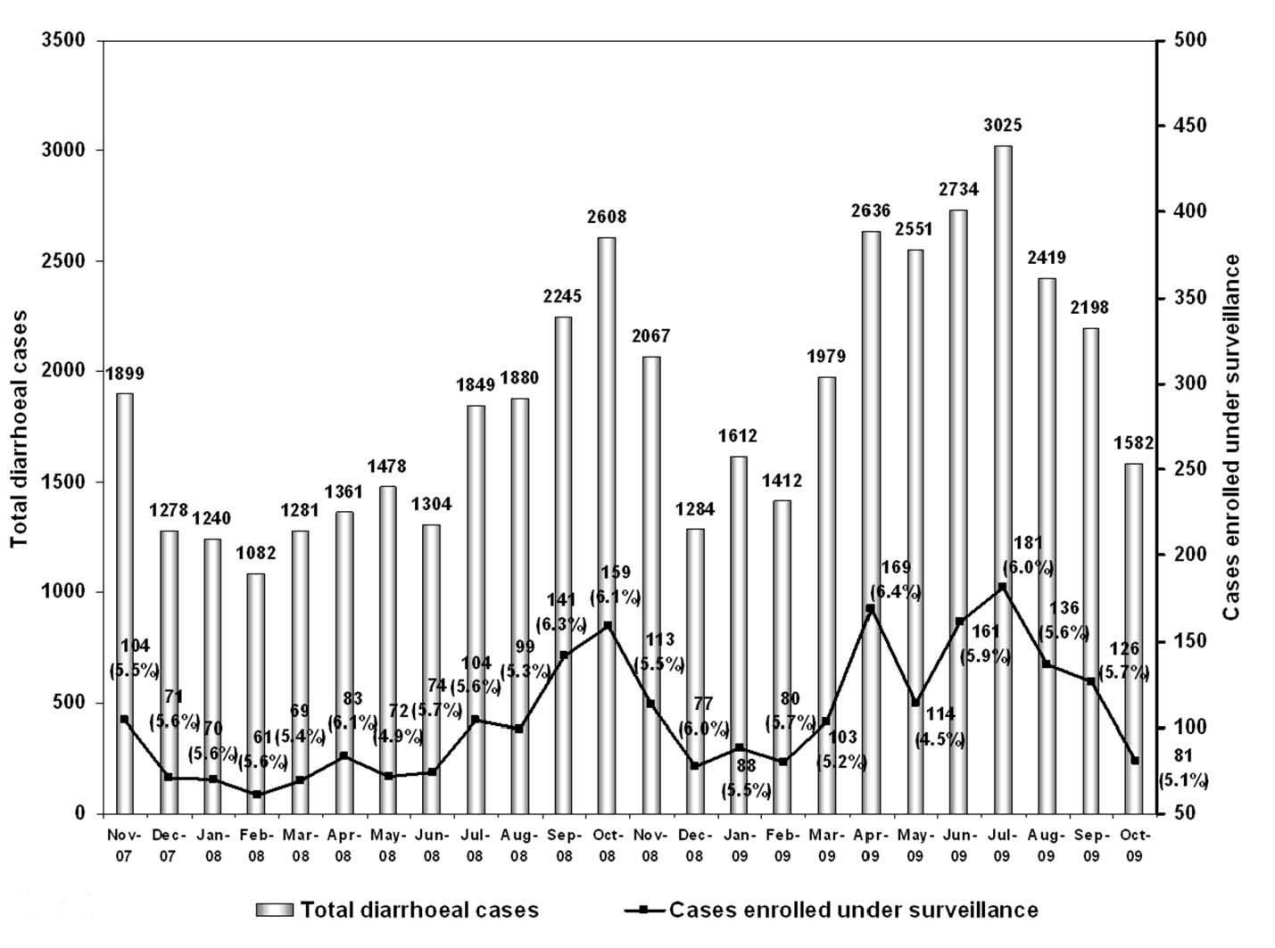
**Month-wise details on number of admitted and enrolled cases in the surveillance**.

**Figure 2:**
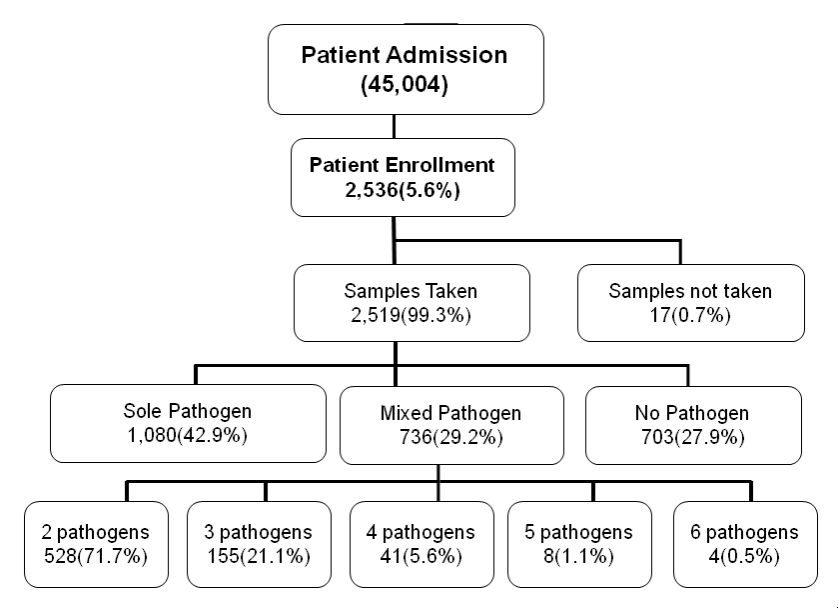
**Flowchart showing the admission rates of diarrhoea, the number of cases enrolled in the active surveillance, sampling details and prevalence status of the pathogens**.

An increasing trend in the hospital admissions of diarrhoea was observed from July to October, 2008 with a peak in October but during the following year 2009, it was during March to July with a peak in July (Fig. 1). As shown in the flow chart (Fig. 2), a total of 2536 (5.6%) cases out of 45,004 patients hospitalized with acute diarrhoea in ID&BGH between November 2007 and October 2009 were enrolled. Faecal specimens were collected from 2519 (99.3%) cases for etiological studies (stool specimen could not be collected from 17 enrolled cases), out of which 2499 (99.2%) were neat stool specimens and 20 (0.8%) were rectal swabs. Among the enrolled patients, 80.9% were from urban areas and 54.3% were male patients (data not shown). Demographic information further revealed that average monthly family income of majority of patients (74.7%) ranging from INR.2500 to 5000 (US$ ~50-100). Most of the patients (74.3%) had access to tap water (corporation or municipal supply) as the main source of drinking water. Of the 2536 enrolled patients, 76.1% presented with acute watery diarrhoea, 20% with loose stool, 3.3% with bloody diarrhoea and 0.6% cases with mucoid diarrhoea. Vomiting was a predominant clinical feature in 77.7% cases and 35% suffered from abdominal pain. Prior to hospital admission, 37.4% received only oral rehydration solution (ORS), 21.7% home available fluid (HAF) only and 26.3% both ORS and HAF. On admission, some dehydration was present in 92% of the cases and it was of severe degree in 8% of the cases. Intravenous fluid was administered to 76.9% of the enrolled patients.

### Deaths among hospital surveillance patients

During the surveillance period, 382 deaths (8/1000) were recorded. Out of 2536 enrolled patients, there were 32 (1.3%) deaths comprising 20 males and 12 females. Only two deaths were children aged 2 years and 9 years, while others were adults between 17 and 90 years. Most of the deceased were admitted within 48 hrs from onset, with acute watery diarrhoea and severe dehydration resulting from increased frequency of stools (4 to 20 times) during their illness. Twenty patients died within 24 hours, 7 patients died within 25-48 hrs and 5 patients died after more than two days of hospitalization. Faecal specimens were collected from 27 patients before they died and different pathogens detected were *V. cholerae *O1 in 6, *Cryptosporidium *spp in 2, Rotavirus in 2, *G. lamblia *in 1, *Salmonella *in 1, *E. histolytica *in 1. Mixed infection with more than one pathogen was found in 4 cases and in 10 cases, no enteric pathogen was detected.

### Detection of bacterial etiological agents

Among bacterial pathogens, the overall isolation rate of *V. cholerae *O1 was highest (26%) followed by EAEC (6.3%), *Shigella *spp (6.1%), *C. jejuni *(4.7%), and ETEC (4.5%) (Table1). *V. cholerae *O1 strains possessed *ctxB *of the classical biotype as determined by mismatch amplification mutation PCR assay (MAMA-PCR). ETEC group comprising isolates harbouring genes encoding heat-labile (LT) or heat-stable (ST) or both prevailed almost in equal proportions (~4%; data not shown). In this study, we could not detect any STEC or EIEC. When the data was further analyzed with only children below 5 years of age, 16.4% of the children were infected with *V. cholerae *O1, which was higher than the infections caused by EAEC (12%), *C. jejuni *(9.3%) and *Shigella *spp (7.9%) (Table 1).

**Table 1:** Age group wise isolation of enteric pathogen

	Age <5 years					
			
Pathogen*	0 - 11 month (n = 245)	12 - 23 month (n = 227)	24 - 59 month (n = 176)	Total Age <5 yr (n = 648)	Age ≥ 5 yr (n =1871)	All Age Group (n = 2519)
	n(%)	n(%)	n(%)	n(%)	n(%)	n(%)
**Bacteria**						
*V. cholerae O1*	22(9)	34(15)	50(28.4)	106(16.4)	548(29.3)	654(26)
*V. cholerae O139*					2(0.1)	2(0.1)
*V. cholerae *non-O1, non-O139	2(0.8)	1(0.4)	1(0.6)	4(0.6)	51(2.7)	55(2.2)
*V. parahaemolyticus*	1(0.4)		2(1.1)	3(0.5)	71(3.8)	74(2.9)
*V. fluvialis*	3(1.2)	7(3.1)	1(0.6)	11(1.7)	44(2.4)	55(2.2)
*Aeromonas *spp.	1(0.4)	2(0.9)	1(0.6)	4(0.6)	21(1.1)	25(1)
*Campylobacter jejuni*	18(7.3)	22(9.7)	20(11.4)	60(9.3)	58(3.1)	118(4.7)
*C. coli*	1(0.4)		1(0.6)	2(0.3)	20(1.1)	22(0.9)
*Shigella *spp	8(3.3)	21(9.3)	22(12.5)	51(7.9)	103(5.5)	154(6.1)
*Salmonella *spp.		1(0.4)	1(0.6)	2(0.3)	21(1.1)	23(0.9)
*EPEC*	11(4.5)	8(3.5)	2(1.1)	21(3.2)	24(1.3)	45(1.8)
ETEC	9(3.7)	13(5.7)	5(2.8)	27(4.2)	87(4.6)	114(4.5)
*EAEC*	32(13.1)	28(12.3)	18(10.2)	78(12)	81(4.3)	159(6.3)
**Virus**						
*Rotavirus*	130(53.1)	134(59)	48(27.3)	312(48.1)	181(9.7)	493(19.6)
*Adenovirus*	37(15.1)	26(11.5)	12(6.8)	75(11.6)	51(2.7)	126(5)
*Norovirus G1*		2(0.9)		2(0.3)	4(0.2)	6(0.2)
*Norovirus G2*	13(5.3)	10(4.4)	6(3.4)	29(4.5)	43(2.3)	72(2.9)
*Sapovirus*	13(5.3)	4(1.8)	4(2.3)	21(3.2)	20(1.1)	41(1.6)
*Astrovirus*	6(2.4)	7(3.1)	5(2.8)	18(2.8)	41(2.2)	59(2.3)
**Parasite**						
*Blastocystis hominis*					11(0.6)	11(0.4)
*Entamaeba histolytica*	8(3.3)	13(5.7)	5(2.8)	26(4)	56(3)	82(3.3)
*Giardia lamblia*	25(10.2)	34(15)	33(18.8)	92(14.2)	189(10.1)	281(11.2)
*Cryptosporidium spp*.	37(15.1)	22(9.7)	12(6.8)	71(11)	87(4.6)	158(6.3)

*STEC and EIEC were negative in all stool specimens

Of the 16 *V. parahaemolyticus *isolates, 3 were identified as O3:K untypable (UT) and others belonged to assorted serotypes. All the 55 non-O1, non-O139 *V. cholerae *strains were confirmed as *V. cholerae *by *ompW *PCR and belong to different serogroups (data not shown). The *V. fluvialis *strains were identified by biochemical tests and confirmed by species-specific *toxR *PCR. Among shigellae, *S. flexneri *was the most common (74.7%), followed by *S. sonnei *(16.2%), and *S. boydii *(7.1%) (data not shown). *S. dysenteriae *type 1 serotype was not detected and also the other serotypes were present in very low numbers (1.9%), which is an unusual trend observed in this study. Among children less than 5 years of age, *S. flexneri *was detected at the level of 5.5%, *S. sonnei *(2.2%) and *S boydii *(0.2%). *S. flexneri*, was mainly represented by serotypes 2a (51%) and 3a (28.7%). Interestingly, 13% of the *S. flexneri *strains remained untypable in the exiting *Shigella *spp serotyping scheme, which is also an emerging trend. In this study, 23 non-typhoidal salmonellae were isolated from diarrhoeal patients, of which, Enteritidis (26%), Weltevreden (13%) are the common serotypes and 7 strains could not be serotyped (data not shown). Altogether, 318 DEC were isolated, of which 126 were associated children <5 year age group. Among DEC, EAEC was high (62%) among children <5 years of age, where as ETEC was more among ≥5 years age group (45.3%) (Table 1).

### Antimicrobial susceptibility

Antimicrobial susceptibility testing was made with *V. cholerae *O1 and shigellae strains as these pathogens dominated the rest. Of the 230 representative strains of *V. cholerae *O1 examined, 47% were resistant to tetracycline (Table 2). Fifty eight per cent of the *V. cholerae *isolates showed reduced susceptibility to ciprofloxacin. However, most of the strains remained susceptible for azithromycin (97.5%), norfloxacin (99.1%), chloramphenicol (98.7%) and neomycin (100%). Multidrug resistance was very high in shigellae as most of them were highly resistant to fluoroquinolones (ciprofloxacin, 90.3%, norfloxacin, 83.1%, and ofloxacin, 81.8%), and nalidixic acid (93.5%). As shown in Table 2, most of the isolates were also resistant to other antimicrobials (40-98%). However, majority of the shigellae were susceptible to ceftriaxone (94.8%).

**Table 2:** Antimicrobial resistance in *V. cholerae *O1 and *Shigella *spp

	Percentage resistance	
Antimicrobial	*Vibrio cholerae *(n = 230)	*Shigella *sp. (n = 154)
Ampicillin	61.7	54.5
Azithromycin	2.5*	41.6
Ceftriaxone	12.3*	5.2
Chloramphenicol	1.3	64.3
Ciprofloxacin	8.7	90.3
Co-Trimoxazole	87.4	94.2
Doxycycline	1.2*	ND
Erythromycin	10.4	98.7
Furazolidone	90.9	91.6
Gentamycin	0.0	ND
Nalidixic acid	93.0	93.5
Neomycin	0.0	ND
Norfloxacin	0.9	83.1
Ofloxacin	7.4*	81.8
Streptomycin	84.9	98.1
Tetracycline	47.0	89.0

Note: * n = 81, ND = Not Done

### Detection of viral etiological agents

Rotavirus was associated with 19.6% of the cases in all age groups and was the major viral agent associated with 48.1% of the infections among children <5 year age group (Table 1). Human Adenovirus (5%), Astrovirus (2.3%), Norovirus (NoV) (3.1%) and Sapovirus (SaV) (1.6%) were also detected but heir prevalence was low. Compared to other enteric viruses, prevalence of Adenovirus was more (11.6%) in children <5 years age group.

### Detection of parasitic etiological agents

Overall, 532 samples were found positive for parasitic infections. Among these, *G. lamblia *was most predominant in 281 (11.2%) cases followed by *Cryptosporidium *sp. (6.3%) and *E. histolytica *(3.3%) (Table 1). The dominance of *G. lamblia *is an emerging trend in Kolkata where amoebiasis has been a dominant parasitic pathogen over many years. The higher age group has shown to be equally susceptible to these parasites as observed in children below 5 years. Although the proportions of infections caused by *Giardia *and *Entamoeba *in children aged below five years were almost equivalent as observed in higher age group (≥5 yrs) of patients with diarrhoea, cryptosporidiosis was more predominant (11%) in children below five years of age.

### Mixed infections and no pathogens

**Figure 3:**
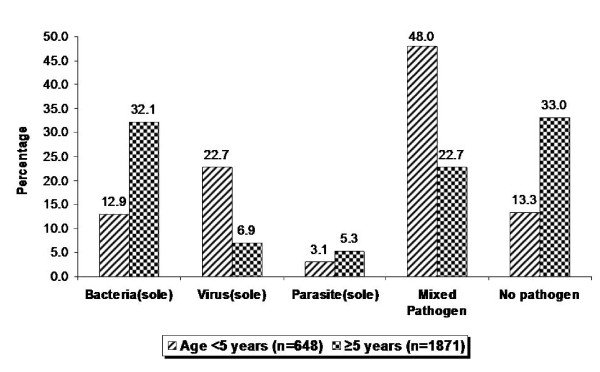
**Comparison of infection status by different enteric pathogens in < 5 and ≥5 age groups**.

Viral pathogens dominated in the <5 years age group while in the ≥5 years age group, bacterial pathogens dominated (Fig. 3). Overall, 29.2% of the 2519 cases showed mixed or polymicrobial infections associated with diarrhoea as shown in Fig. 2. In children <5 years of age, 48% of the cases were associated with mixed infections while in ≥5 year age group, mixed infections were seen in 22.7% of the cases (Fig. 3). We examined the mixed infections in two dominant pathogens namely *V. cholerae *O1 and rotavirus (Table 3). Among 654 cholera cases, sole *V. cholerae *infection was observed in 377 (57.6%) of the cases. Of the 493 cases positive for rotavirus, 285 (57.8%) were coinfected with other pathogens. Co-infection of parasites with *V. cholerae *O1 and Rotavirus was high among diarrhoeal patients (Table 3). Further, in 27.9% of the cases, no potential pathogen was detected but in children below 5 years of age, the proportion of no potential pathogen was less (13.3%).

**Table 3:** Observation of mixed pathogens with *V. cholerae *O1 and Rotavirus infection from 2519 acute diarrhoeal samples

Main Pathogen With other pathogen	*V. cholerae *O1 **(Sole pathogen = 377**, **Mixed pathogen = 277)**	Rotavirus **(Sole pathogen = 208**, **Mixed pathogen = 285)**
	
	n (%)	n (%)
*V. cholerae*^1^	-	46 (16.1)
Shigellae	12 (4.3)	11 (3.9)
DEC^2^	68 (24.5)	80 (28.1)
Other enteric bacteria^3^	70 (25.3)	57 (20)
Rotavirus	41 (14.8)	-
Other virus^4^	36 (13)	98 (34.4)
Parasites^5^	126 (45.5)	117 (41.1)

Note:

1. *V. cholerae *O1, *V. cholerae *O139 and *V. cholerae *no- O1, no- O139

2. EPEC, ETEC (LT), ETEC (ST), ETEC (LT, ST) and EAEC

3. *V. parahaemolyticus*, *V. fluvialis*, *Aeromonas spp*., *C. jejuni*, *C. coli *and *Salmonella*

4. Adenovirus, Norovirus GI, Norovirus GII, Sapovirus and Astrovirus

5. *B. hominis*, *E. histolytica*, *Giardia lamblia *and *Cryptosporidium sp*

**Figure 4:**
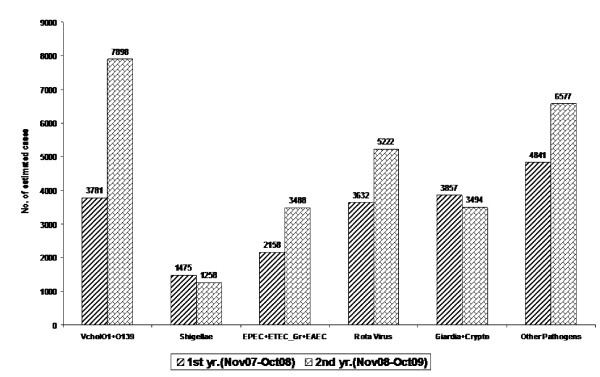
**Estimation of infections by different enteric pathogens in two consecutive years**.

The estimated number of cholera cases in the second year was as high 7898 cases which was much higher than that seen in the first year (3781). At the ID&BGH, choler clearly is the most important etiology in both the years followed by Rotavirus (Fig. 4). Patients infected with Rotavirus and DEC, were also more in the second year. However, shigellae, *Giardia *and *Cryptosporidium *were lesser during second year of surveillance.

### Statistical analysis

The age was classified into 8 categories, in which 245 (9.7%), 261 (10.4%), 163 (6.5%) 202 (8.0%), 666 (26.4%), 492 (19.4%), 293 (11.6%) and 197 (7.8%) cases fall in the age groups of <1, >1-2, >2-5, >5-14, >14-30, >30-45, >45-60 and >60 years, respectively. The MLR analysis showed that ≤2 years age group was significantly less infected by *V. cholera*e O1 (p < 0.001). Whereas rotavirus was significantly higher in the <2 years (p < 0.001) and >2-5 years (p < 0.001) age groups. *G. lamblia *infection was present in up to 5 years (p < 0.001) and >5-30 years (p = 0.001) age groups. Shigellosis was found in children with 1- 5 years age group (p > 0.05), when comparison referred to more than sixty years of age enrolled subjects in this study (Table 4). The relationship between the risk dependent variable and each of the categorical explanatory variable are shown in Table 4.

**Table 4:** Multinomial Logistic Regression Models exploring significant risk age group of predominant enteric pathogenic infection at IDH, Kolkata (November 2007-October 2009)

Age in Years	Enteric pathogens	B	OR (95% CI)	p-value
**Below 1 yrs**	*V. cholerae *O1	-1.31	0.27(0.16-0.46)	0.000*
	*Rotavirus*	2.46	11.72(6.73-20.41)	0.000*
	*Shigella*	-0.81	0.44(0.18-1.08)	0.073
	*Giardia lamblia*	0.87	2.39(1.09-5.24)	0.030*
**1 to 2 yrs**	*V. cholerae *O1	-0.74	0.48(0.30-0.76)	0.002*
	*Rotavirus*	2.42	11.28(6.54-19.45)	0.000*
	*Shigella*	0.32	1.38(0.70-2.73)	0.354
	*Giardia lamblia*	1.38	3.99(1.89-8.41)	0.000*
**>2 to 5 yrs**	*V. cholerae *O1	0.32	1.38(0.88-2.17)	0.162
	*Rotavirus*	1.05	2.85(1.55-5.25)	0.001*
	*Shigella*	0.66	1.93(0.95-3.93)	0.070
	*Giardia lamblia*	1.38	3.96(1.80-8.71)	0.001*
**>5 to 14 yrs**	*V. cholerae *O1	0.36	1.43(0.93-2.19)	0.101
	*Rotavirus*	-0.05	0.95(0.48-1.87)	0.885
	*Shigella*	0.18	1.20(0.57-2.49)	0.636
	*Giardia lamblia*	1.81	6.11(2.90-12.88)	0.000*
**>14 to 30 yrs**	*V. cholerae *O1	0.23	1.26(0.89-1.80)	0.195
	*Rotavirus*	-0.01	0.99(0.57-1.72)	0.977
	*Shigella*	-0.68	0.51(0.26-0.99)	0.040*
	*Giardia lamblia*	0.79	2.20(1.08-4.51)	0.031*
**>30 to 45 yrs**	*V. cholerae *O1	-0.15	0.86(0.59-1.26)	0.444
	*Rotavirus*	0.06	1.06(0.60-1.87)	0.845
	*Shigella*	-0.44	0.65(0.33-1.28)	0.209
	*Giardia lamblia*	0.70	2.01(0.96-4.21)	0.064
**>45 to 60 yrs**	*V. cholerae *O1	0.06	1.07(0.71-1.60)	0.756
	*Rotavirus*	-0.01	0.99(0.53-1.83)	0.965
	*Shigella*	0.00	1.00(0.50-2.02)	0.992
	*Giardia lamblia*	0.70	2.02(0.93-4.41)	0.077
**>60 yrs**		Reference category		

*statistically significant

### Seasonality

**Figure 5:**
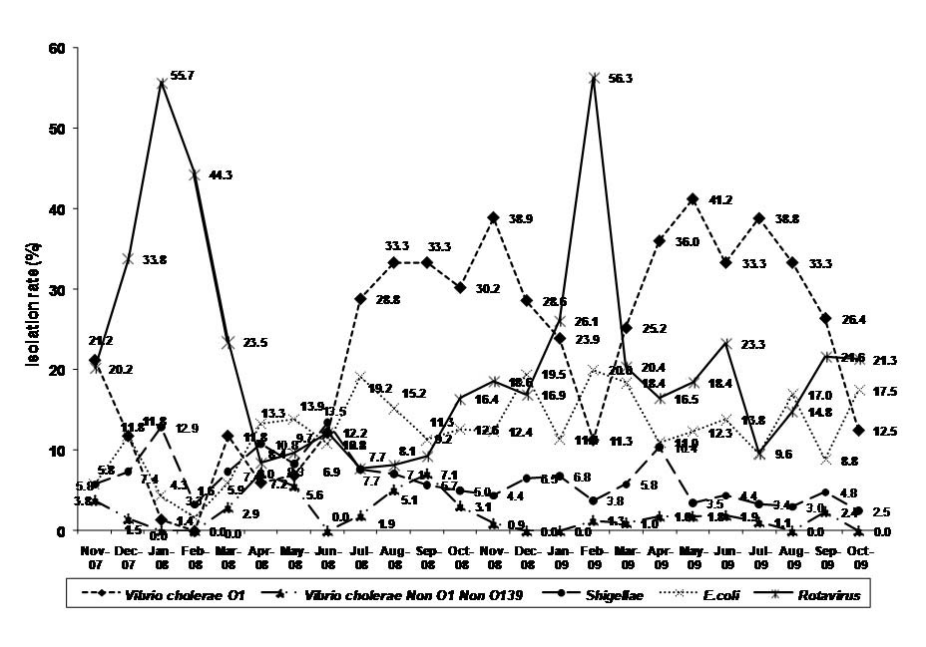
**Seasonality of predominant diarrhoeal pathogens in this hospital based surveillance**.

Cholera and rotavirus mediated diarrhoea had distinct seasonality with peaks during July-September (monsoon) and December-February (winter), respectively (Fig. 5). In 2008, unusual *V. cholerae *infections were observed during November-December may be due to increased temperature and humidity in Kolkata. There was no remarkable seasonal trend for other bacterial or viral pathogens. Among parasitic pathogens, *G*. *lamblia *was detected constantly throughout the year, whereas the prevalence of *Cryptosporidium *sp. and *E. histolytica *showed minor seasonal variations.

## Discussion

Rotavirus and *V. cholerae *O1 were the most common enteric pathogens causing diarrhoea that required hospitalization with the former dominating during winter months and the latter in the other months. This trend of pathogen dominance has remained unchanged for many years in the hospital setting in Kolkata with occasional interannual variations [4,5]. The proportions however seem to have changed because an etiological study on hospital in patients with acute diarrhoea in Kolkata at the same hospital from July 1979 to October 1981 showed that *V. cholerae *O1 was detected in 30.3% of the cases while the proportion of rotavirus was 7.6% [4]. A trend which is distinctly different from the past was the incidence of cholera in <2 years age group. Not only is the trend seen among hospitalized cases but is also reminiscent in the community as revealed by a population based study in an urban slum setting in Kolkata, where the incidence of cholera (6.2/1000) was highest in children <5 years [3]. In this study, significant association of *V. cholerae *infection was detected in children <1 to 2 years age group. Likewise, it was found that young children bear the greatest burden of cholera in Jakarta (Indonesia), Beira (Mozambique), and Bangladesh [3,6]. This is an important shifting trend given that WHO previously recommended that cholera should be suspected among those more than 2 years of age who have acute watery diarrhoea and severe dehydration if cholera is endemic in the local area [7]. Cholera in young children in an endemic area was first reported from Kolkata in 1992 necessitating a change in the WHO recommendation [8].

In the past, cholera predominantly occurred during the summer months (April-June) in Kolkata but there appears to be a shift in its seasonality from summer to monsoon. Cholera is a climate responsive disease and subtle changes in climatic parameters could have a discernible difference in its incidence. A recent study on the pattern of cholera outbreaks during 1998 to 2006 in Kolkata, has shown a statistically significant relationship between the time series for cholera and 'chlorophyll-a' concentration and rainfall anomalies [9]. The current *V. cholerae *O1 belonged to the newly reported El Tor variant [10] because all the representative strains examined had the *ctxB *gene of the classical type. The El Tor variant strains became prevalent in Kolkata since 1991 [11]. *V. cholerae *non-O1, non-O139 mediated infections appear to be increasing compared to previous years [12] and in some Indian studies, the detection rate among diarrhoeal patients was as high (25.8%) [13].This is another emerging trend which needs to be carefully monitored.

The yearly estimated number of cholera cases at the ID&BGH brings out the reality of underestimation of cholera and other enteric infections at the National level. The 2008 National Health Profile, a compilation of the Central Bureau of Health Intelligence reports that in the year 2008 there were 11231038 episodes of diarrhoea and 2680 cases of cholera in India [14]. The report does highlight the limitation of the completeness of the data due to variety of reasons. However, from the estimate made in this study, one infectious diseases hospital in Kolkata had larger number of cholera cases in both the years (Fig. 5) than the national number of cases of cholera reported. To combat a disease, a true understanding of the burden of the disease is important to implement realistic interventions and assess the significance of these interventions to understand how they impact on the reduction of the number of cholera cases.

Shigellae infection was significantly associated with older age group (>45 years). Though *S. flexneri *and *S. sonnei *were commonly associated with diarrhoea, *S. dysenteriea *was rarely isolated in this study. Increase in isolation rates of untypable *S. flexneri *and *S. sonnei *are the new propensities of shigellosis, which is different from a previous study in Kolkata [4]. This is the first study in Kolkata that has included the common pathogroups of DEC in the surveillance. Prevalence of DEC, mainly, the ETEC and EPEC was much lower in this study than seen in Bangladesh and Ghana, where their incidence was 12% and 14.8%, respectively [15,16]. Among EPEC mediated diarrhoea, atypical EPEC harbouring *eae *gene was isolated more frequently (77.7%) than the typical EPEC (22.3%) (with *eae *and *bfpA *genes, data not shown), which is an emerging trend in many developing countries [17]. In this study, the prevalence of *C. jejuni *mediated infection was more than the *C. coli *and this trend appears common in many countries [18].

As reported in many cholera endemic regions, tetracycline resistance was high among *V. cholerae *O1 in Kolkata [19,20]. Increase in the reduced susceptibility of *V. cholerae *O1 for ciprofloxacin is a great concern. Considering these trends, azithromycin is being used in the ID&BGH for the treatment of cholera patients. Emergence of MDR in shigellae is a global problem [21]. Except for ceftriaxone, most of the *Shigella *strains in this study were resistant for more than 10 antimicrobials. Shigellosis in this part of the world is likely to be an untreatable disease if this pattern of multiple drug resistance continues. In several findings, the emergence of multidrug resistant strains of *V. cholerae *O1 and shigellae has been reported due to different genetic factors including transfer of plasmids, integrons and allelic variation in the specific genes. In developing countries, fluoroquinolones are extensively used for the treatment of many infectious diseases (monotherapy coupled with inadequate dosage) including diarrhoea and perhaps this is one of the factors for the emergence of multidrug resistance among potential enteric pathogens.

The etiological role of rotavirus in hospitalized diarrhoea cases indicated that infections were mostly associated with acute watery diarrhoea among hospitalized patients in <5 years age group (48.1%) than in the higher age group (9.7%) and this association was statistically significant. This study also revealed that rotavirus infections occurred both as the sole pathogen (8.3%) as well as in association with other pathogens (11.3%) mostly with parasites, and to a lesser extent with other viruses or bacteria. Adenovirus that belong to subgenus F serotypes (40/41) were detected infrequently (5%) among children aged <5 years than adults, with a higher frequency as mixed infections.

Similar to Adenovirus, Norovirus rank next to rotavirus and they have gained widespread recognition as the major cause of food borne and epidemic associated viral gastroenteritis in several studies [22,23]. NVGI was detected occasionally (0.2%) mostly in >5 year age group, with much lower incidence than NVGII, which was detected also intermittently (2.9%) in all age groups, mostly as mixed infections. The frequency of Astrovirus and Sapovirus was low in this study (2.3 and 1.6%, respectively). Reports from developed and developing countries indicate that prevalence of Astrovirus and Sapovirus among diarrhoeal patients is constantly low. Our figures are analogous to prevalence of Astrovirus infection that was reported to vary from 3 to 16% among hospitalized children with diarrhoea [24,25].

Parasitic infections are very common in children in developing countries. *G. lamblia *showed the significant high risk age group as >5-14 yrs, though the lesser age groups were also involved. The detection rate of *Cryptosporidium *spp is comparable with other investigations [16,26]. Two major observations recorded in this study were the infection of *Giardia *in the adult patient group of up to 20 years and significant association of *Giardia *as co-infection with other pathogens. While comparing the rate of *Giardia *infections with that of preceding years in the same area among similar group of patients, it was observed that there is a slow but obvious change in the pattern of infection with time [27]. Prevalence of *E. histolytica *was low in this study and this may be related to the current use of chloroquine under the malaria eradication program of Ministry of Health and Family Welfare. In addition, this factor might have offered indirect support for the increase in *Giardia *infections.

Majority of the hospitalized diarrhoeal patients were infected with more than one pathogen. Polymicriobial infection (20-50%) seems widespread in many developing countries [28]. This trend in diarrhoea is a clear indication that the source of infection may be related to grossly contaminated food and water, as most of the patients were from low-income group and living in unhygienic environments. Despite covering about 24 enteric pathogens, in 27.9% of the cases we could not identify any pathogen and this trend also seems common in many diarrhoea endemic regions [29,30]. If a vaccine is available against *V. cholerae *O1, rotavirus and shigellae, the overall hospitalization due to diarrhoea can be considerably reduced. Rotavirus and cholera vaccines are now available as prescription product in India for the first time after a hiatus of 30 years. Not much progress has, however, been made with a *Shigella *vaccine. It would be interesting to see how these vaccines would ameliorate the burden of enteric infections in settings like Kolkata and other diarrhoea endemic areas in India.

## Conclusions

Increase in *V. cholerae *O1 infection among <2 years age group, resistance of *V. cholerae *O1 to tetracycline, rise of untypable *S. flexnerii*, higher proportion of atypical EPEC and *G. lamblia *and polymicrobial etiology are some of the emerging trends observed in this diarrhoeal disease surveillance.

## Methods

The Infectious Diseases and Beliaghata General Hospital (ID&BGH), in Kolkata, a 770 bedded hospital, provides treatment for about 20,000 to 25,000 hospitalized patients with acute diarrhoea annually. In the present systematic active surveillance, every fifth patient with diarrhoea or dysentery without other associated illness on two randomly selected days of the week was enrolled as study subjects from cases admitted at the ID&BGH. This study was conducted between November 2007 and October 2009. The dehydration status of each diarrhoea case was classified as no, some or severe dehydration according to WHO guidelines. The clinical, demographic and laboratory data was checked manually and entered into pre-designed data entry proforma developed in visual basic with inbuilt entry validation checking facilitated programme in structure query language (SQL) server by dual entry method by trained data entry professionals. Data was randomly checked and matched to derive consistency and validity for analysis. The edited data was exported and a final analysis was performed using the SPSS.17.0 software (SPSS Inc., Chicago, IL, USA).

**Figure 6:**
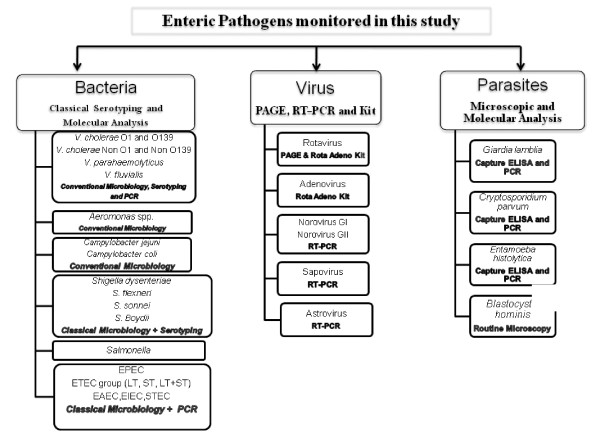
**Chart showing the list of bacterial, viral and parasitic pathogens that were examined during the active hospital based diarrhoea surveillance**.

This study was approved by the duly constituted Institutional Ethics Committee (IEC). As per the recommendation of IEC, individual informed consent was obtained from each patient enrolled in this study and confidentiality was maintained. Faecal specimens were collected in McCartney bottles using sterile catheters or as rectal swabs in Cary Blair medium and were examined within 2 hrs for 24 enteric pathogens comprising bacterial, viral and parasitic pathogens using a combination of conventional, immunological and molecular methods (Fig. 6). PCR targeting *omp*W and *toxR *were performed for the species confirmation of *V. cholerae *and *V. fluvialis*, respectively [31,32]. Confirmed strains of *V. parahaemolyticus*, *Shigella *spp and *Salmonella spp *were serotyped using commercially available antisera (Denka Seiken, Tokyo, Japan, BioRad, Marnes-la-Coquette, France). *V. cholerae *strains were serotyped using antisera prepared in NICED. Representative strains of *V. cholerae *O1 were examined by MAMA-PCR to determine the type of cholera toxin B subunit gene (*ctxB*) [33]. Three different lactose-fermenting colonies isolated from each sample were picked from MacConkey agar plate and included in the multiplex PCR assay for the detection of different DEC that include enterotoxigenic *E. coli *(ETEC, inclusive of both heat-labile and heat-stable enterotoxin producers), enteropathogenic *E. coli *(typical and atypical EPEC) and enteroaggregative *E. coli *(EAEC) [34]. Simplex PCR was also performed for the detection of enteroinvasive *E. coli *(EIEC) and Shiga toxin-producing *E. coli *(STEC) [35,36].

Antimicrobial susceptibility testing was performed by disk diffusion (Kirby- Bauer method) using commercially available disks (Becton Dickinson Co., Sparks, MD, USA) with interpretation stipulated by the Clinical and Laboratory Standard Institute [37]. Two hundred and thirty representative (one third from the total number of strains) *V. cholerae *O1 strains covering all the months and all the *Shigella *strains were included in the testing. Rotavirus was detected by polyacrylamide gel electrophoresis and silver staining [38]. Norovirus [Group I and II (NVGI and NVGII)], Sapovirus and Astrovirus were detected by RT-PCR using random primers for reverse transcription and specific primers for polymerase chain reaction [24,39]. Different viruses were detected according to the appropriate amplicon sizes observed in agarose gels stained with ethidium bromide. Adenovirus was detected by the commercially available RotaAdeno VIKIA kit (biomereux, France), which is a qualitative test-based on immunochromatography in lateral flow format [40]. For detection of enteric parasites, faecal samples were processed separately for microscopic and molecular analysis. For microscopic analysis, the samples were first concentrated using formalin ethyl acetate concentration method [41] and an aliquot of each sample was preserved in 10% formalin and stored at 4°C for subsequent use. Aliquots of fresh stool specimens were also preserved at -80°C for ELISA and PCR assays. All the faecal samples were screened using a highly sensitive antigen capture ELISA (Tech Lab, Blacksburg, USA) and PCR for the detection of *Giardia lamblia*, *Cryptosporidium parvum *and *Entamoeba histolytica*. Faecal samples were processed by microscopy using iodine wet mount staining and trichome staining procedure for *Blastocystis hominis *[42].

Using the surveillance data, an estimate of the total number of cases specific for each pathogen in two consecutive years was extrapolated. From the monthly enrolled cases, the isolation rate of different pathogens was calculated for that particular month. An estimate of total number of cases with particular pathogen for a particular month was then extrapolated by multiplying the total admitted cases with particular isolation rate of the pathogenic with an assumption that similar isolation rate would be among non-enrolled cases. In this way, pathogen-specific total number of yearly estimated cases was calculated.

The risk age group was also explored for predominant enteric pathogens such as *V. cholerae *O1, Rotavirus, shigellae and *G. lamblia *by Multinomial Logistic Regression (MLR) analysis [43,44]. This analysis helps to determine the likelihood age of the patient associated with any enteric pathogen. The age groups were classified into 8 categories *viz*. <1 year, 1-2 years, >2-5 years, >5-14 years, >14-30 years, >30-45 years, >45-60 years and >60 years and were coded from 1 to 8, respectively. Infection caused by an enteric pathogen was coded as '1' for the pathogen present and '2' for its absence. The extreme values of the classified age group was fixed as a reference category.

## Competing interests

The authors declare that they have no competing interests.

## Authors' contributions

GBN conceived of the study, KO and YT participated in the design of this study, MKB and MG coordinated collection of specimens, maintenance of clinical data and management of patients, TR carried out bacterial screening, TK carried out virus screening, SG and DRS carried out screening of parasites, KR and BM coordinated data management and performed statistical analysis. All authors read and approved the final manuscript.
